# P-1718. Predicting Mortality in Candidemia: The Role of SOFA Score and Age in a Multivariate Model

**DOI:** 10.1093/ofid/ofaf695.1889

**Published:** 2026-01-11

**Authors:** Thaís de Paula Guimarães, Sinval Silva, Amanda F Negreiros, Giulia M C Ardisson, Henrique L V Santos, Eduardo F Sad, Rogério Pereira, Ana Paula Lafdeira, Bráulio R G M Couto

**Affiliations:** Hospital Felício Rocho, Belo Horizonte, Minas Gerais, Brazil; Hospital Felício Rocho, Belo Horizonte, Minas Gerais, Brazil; Hospital Felício Rocho, Belo Horizonte, Minas Gerais, Brazil; Hospital Felício Rocho, Belo Horizonte, Minas Gerais, Brazil; Hospital Felício Rocho, Belo Horizonte, Minas Gerais, Brazil; Hospital Felício Rocho, Belo Horizonte, Minas Gerais, Brazil; Hospital Felício Rocho, Belo Horizonte, Minas Gerais, Brazil; Biobyte Brasil, Belo Horizonte, Minas Gerais, Brazil; AMECI – Associação Mineira de Epidemiologia e Controle de Infecções, Belo Horizonte, Minas Gerais, Brazil

## Abstract

**Background:**

The objective of our study is to answer two questions: 1) what is the mortality due to Candidemia in a general hospital of a developing country? 2) is it possible to build an accurate model to predict mortality after Candidemia?

**Table 1:**
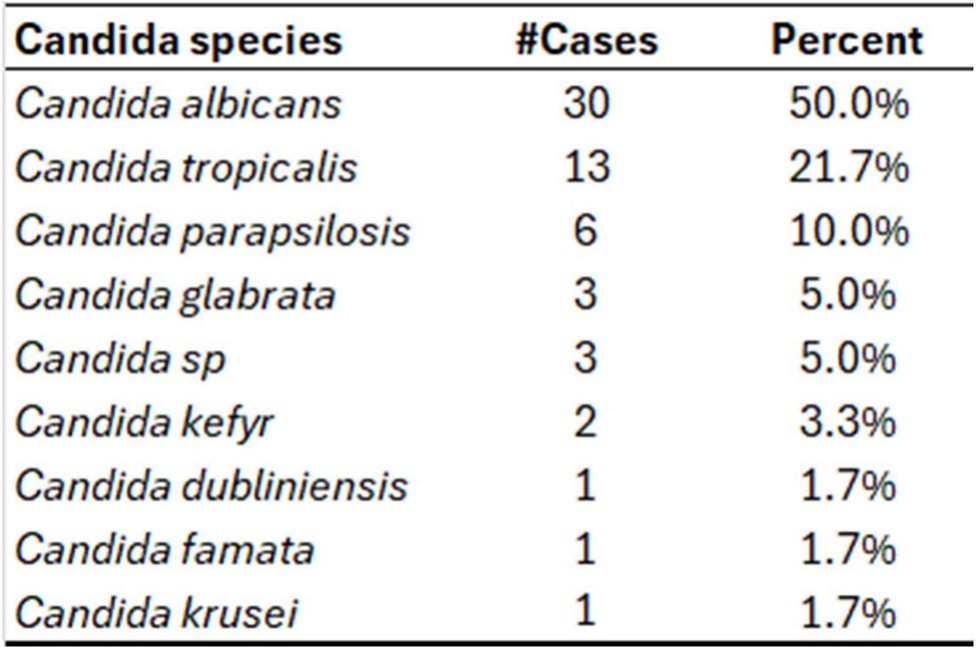
Candida species causing Candidemia. The vast majority of candidemia cases (82%) were caused by just three Candida species: albicans, tropicalis, and parapsilosis.

**Table 2:**
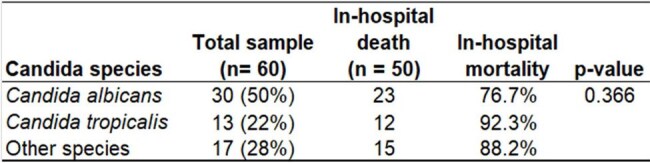
In-hospital mortality by Candida species. There was no significant difference in mortality among the Candida species.

**Methods:**

This single-center retrospective cohort study was conducted between Nov/2019-Oct/2024, involving patients with bloodstream infection caused by Candida species admitted to an Intensive Care Unit of a hospital in Belo Horizonte, a city with approximately three million inhabitants in Brazil. Occurrence of in-hospital mortality was calculated using point estimation and a 95% confidence interval. Subsequently, univariate and multivariate analyses were performed to identify factors associated with in-hospital mortality.

**Table 3:**
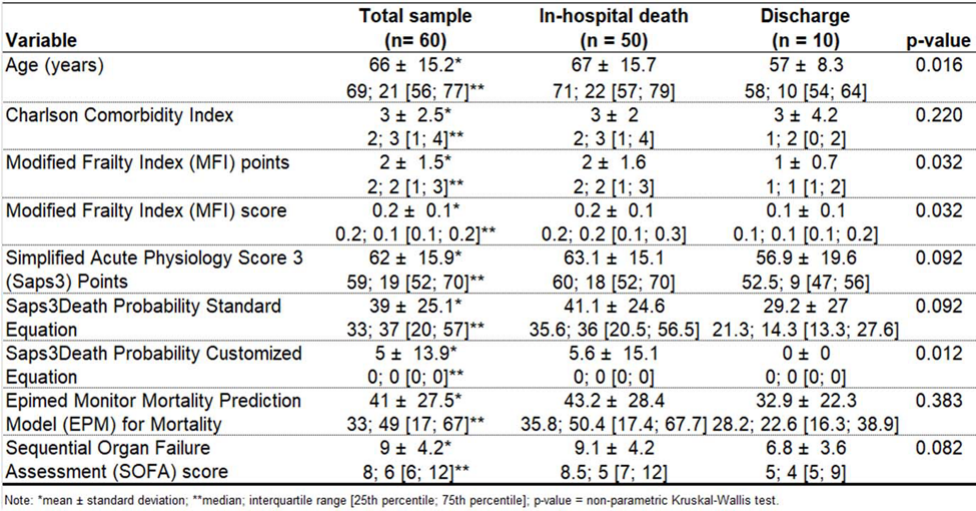
Univariate analysis of quantitative variables to identify factors associated with in-hospital death due to candidemia. Patients who progressed to death were significantly older and had a greater intrinsic severity of illness.

**Table 4:**
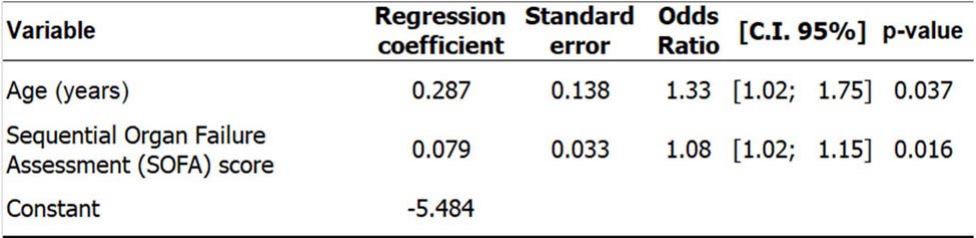
Multivariate analysis (using logistic regression) to identify factors associated with in-hospital mortality due to candidemia. In the multivariate analysis, age and SOFA score independently explain why some patients progress to death and others are discharged.

**Results:**

A sample of 60 patients with bloodstream infection caused by *Candida* (candidemia) was evaluated between November 2019 and October 2024: 50% were between 70 and 80 years old, with a mean age of 66 ±15 years. The vast majority of candidemia cases (82%) were caused by just three Candida species: *albicans, tropicalis*, and *parapsilosis* (Table 1). For the treatment, 56% of the cases received Anidulafungin, 29% Fluconazole, 7% Liposomal Amphotericin B, 5% Micafungin, and 2% Voriconazole. In total, 50 patients progressed to in-hospital death, resulting in a global mortality rate of 83% (95% CI = [71.5%; 91.7%]). There was no significant difference in mortality among the Candida species (Table 2). Patients who progressed to death were significantly older and had a greater intrinsic severity of illness (Table 3). In the multivariate analysis (Table 4), age and SOFA score independently explain why some patients progress to death and others are discharged. The ROC curve considering the logistic regression model for in-hospital death prediction presented a fair predictive ability, with an area under the curve = 0.75 (95% CI = [0.63; 0.87]).

**Conclusion:**

Single-center Brazilian ICU study found 83% in-hospital candidemia mortality. Older age and SOFA score predicted death independently. Logistic model showed fair mortality prediction (AUC 0.75) for risk stratification, highlighting candidemia's burden and the importance of age/severity in prognosis.

**Disclosures:**

All Authors: No reported disclosures

